# Assessment of the proliferative and angiogenic effects of the synthetic cannabinoid (R)-5-fluoro ADB on human cerebral microvascular endothelial cells 

**DOI:** 10.22038/IJBMS.2023.71819.15605

**Published:** 2024

**Authors:** Laith Naser AL-Eitan, Saif Zuhair, Iliya Yacoub Khair, Mansour Abdullah Alghamdi

**Affiliations:** 1 Department of Biotechnology and Genetic Engineering, Jordan University of Science and Technology, Irbid 22110, Jordan; 2 Department of Anatomy, College of Medicine, King Khalid University, Abha 61421, Saudi Arabia; 3 Genomics and Personalized Medicine Unit, College of Medicine, King Khalid University, Abha 61421, Saudi Arabia

**Keywords:** Brain, Cannabinoids, Endothelial cells, Glycogen synthase kinase 3 beta, Vascular endothelial growth - factors

## Abstract

**Objective(s):**

The process of vascular formation, also known as angiogenesis, primarily relies on endothelial cell proliferation, migration, and invasion. In recent years, it has been discovered that synthetic cannabinoids (SCs) may potentially impact angiogenic processes within the body. We evaluated the impact of the synthetic cannabinoid (R)-5-Fluoro-ADB on the proliferation rate and angiogenesis in Human Cerebral Microvascular Endothelial Cells (hBMECs).

**Materials and Methods:**

hBMECs were treated with (R)-5-Fluoro-ADB and investigated for cell viability, migration rate, and tube-like structure formation. Furthermore, angiogenic-related proteins including Angopoitein-1 and -2, and Vascular Endothelial Growth Factors (VEGF) were examined on mRNA and protein levels.

**Results:**

The results showed a notable rise in the rate of proliferation (*P*-value<0.0001) of HBMECs induced by (R)-5-Fluoro-ADB. The angiogenic capacity of HBMECs was also enhanced between 0.001 μM to 1 μM (R)-5-Fluoro-ADB. Moreover, an increase in the levels of ANG-1, ANG-2, and VEGF mRNA and protein, as well as elevated phosphorylation rate of GSK-3β, were observed across various concentrations of (R)-5-Fluoro-ADB.

**Conclusion:**

Our results suggest an innovative approach in pharmacology for addressing a range of conditions linked to angiogenesis. This approach involves precise targeting of both cannabinoid receptors type-1 and -2. To achieve this, specific agonists or antagonists of these receptors could be employed based on the particular characteristics of the diseases in question.

## Introduction

Angiogenesis is described as a physiological process characterized by the growth and formation of new vasculatures and capillaries. This intricate process involves multiple cellular stages, including cell proliferation, migration, and invasion (1). Capillaries have a critical function in facilitating the exchange of metabolites and nutrients, as they are present near all body tissues with an active metabolism. The metabolic activity of tissue and angiogenesis are closely intertwined, thereby implying that any alterations in metabolic activity can result in corresponding changes in capillary formation (2). In brain angiogenesis, a correlation exists between increased metabolism, capillary formation, and the improvement of memory and learning abilities, surpassing the role of neurogenesis (3). In recent years, the significance of angiogenesis as a critical therapeutic target has been acknowledged, owing to its pivotal role in either exploiting diseases or inhibiting their progression (4). Peripheral arteries and heart diseases, for instance, can be prevented by stimulating angiogenesis, while inhibiting angiogenesis can reduce cancer development, as it plays an essential role in promoting the transformation of tumors from a benign state to a malignant one through metastasis (5). The regulation of gene expression for multiple proteins directly influences angiogenesis.

Vascular Endothelial Growth Factor (VEGF), Angiopoietin-1 (ANG-1), and angiopoietin-2 (ANG-2) are commonly studied potential angiogenic growth factors that affect angiogenesis and vascular development. VEGF enhances vascular permeability and cell migration due to its mitogenic and anti-apoptotic effects on endothelial cells, thereby contributing to the regulation of both normal and pathological angiogenesis (6). ANG-1 and ANG-2 serve as significant angiogenic regulators with contrasting roles. ANG-1 exhibits major proangiogenic characteristics and is regarded as vascular-protective, as it inhibits vascular inflammation, suppresses plasma leakage, and contributes to the prevention of endothelial cell death (7). On the contrary, ANG-2 destabilizes vascular angiogenesis, despite being activated through the same receptor as ANG-1 (8). 

Glycogen synthase kinase-3 (GSK-3) is an enzyme involved in numerous cellular processes, including the regulation of glycogen synthesis, cell signaling, and gene expression. It functions as a serine/threonine protein kinase primarily known for its role in phosphorylating and inhibiting glycogen synthase, thereby impacting glycogen metabolism. (9). GSK-3 consists of two isoforms: GSK-3α and GSK-3β, encoded by distinct genes. Both isoforms share similar functions and are widely expressed across various tissues and organs (10). Notably, GSK-3β exhibits high expression levels in multiple regions of the mammalian brain (11), significantly influencing the development of Alzheimer’s disease (10, 11). The role of GSK-3β as a regulator of angiogenesis has been established by its modulation of crucial signaling pathways, encompassing the Wnt/β-catenin pathway and the PI3K/Akt pathway. These pathways are crucial for diverse aspects of angiogenesis, encompassing functions like endothelial cell proliferation, migration, and the potential to augment tube formation (12-14). Recent medical studies suggest that inhibiting GSK-3 expression could offer a promising therapeutic approach for cancer inhibition and treatment (13). 

**Figure 1:**
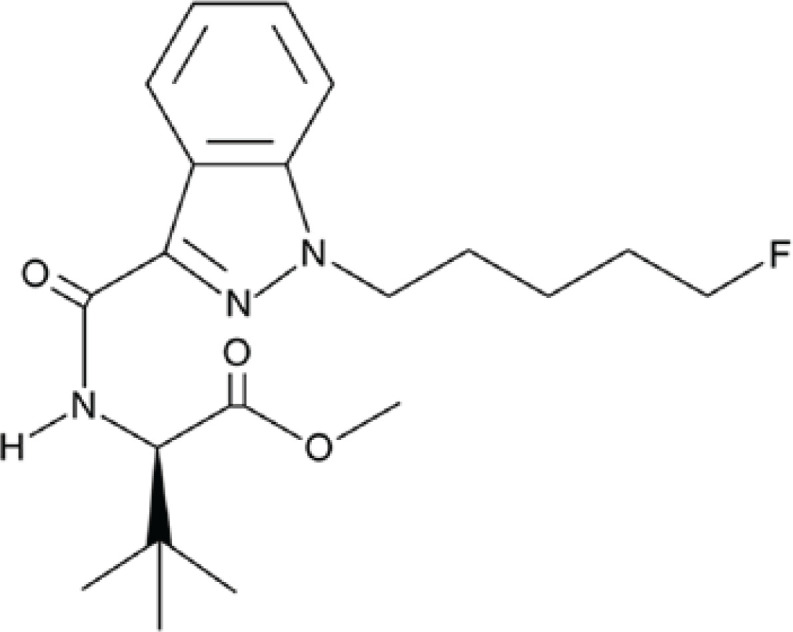
Chemical structure of (R)-5-fluoro ADB

Synthetic cannabinoids have been extensively studied in research pertaining to angiogenesis and vascular formation. In general, cannabinoids, whether natural or synthetic, directly interact with the cannabinoid receptors of the endocannabinoid system, which regulates various functions throughout the body (15). The level of interaction varies among different cannabinoids, with synthetic cannabinoid substances exhibiting higher potency compared to natural cannabinoids and acting as full agonists at endocannabinoid system receptors (16). Among the extensively studied receptors are cannabinoid receptors type 1 and type 2, which play significant roles in regulating intracellular processes and subcellular localization. Cannabinoid receptor type 1 (CB1) is involved in processes related to the brain and nervous system, including synapse formation and plasticity, while cannabinoid receptor type 2 (CB2) is associated with immune cells. (15). The synthetic (R)-5-Fluoro-ADB (C_20_H_28_FN_3_O_3_) also known as (R)-5-fluoro MDMB-PINACA, binds to both CB1 and CB2 cannabinoid receptor (Figure 1) (17). Synthetic cannabinoids (SCs) were initially synthesized for pharmacological purposes, aiming to explore their effects on the endocannabinoid system. However, they have unfortunately been illegally produced by suspicious laboratories (18). (R)-5-Fluoro-ADB is considered one of the most dangerous drugs, associated with severe symptoms such as psychosis, vomiting, mydriasis, confusion, and, in some cases, even death through inhalation (19). Consequently, the (R)-5-Fluoro-ADB drug has been banned in countries such as Japan (20). 

In the present study, we hypothesized that the synthetic cannabinoid (R)-5-Fluoro-ADB could modulate the endothelial cells’ viability and brain cells’ angiogenesis rate. Furthermore, we investigated the phosphorylation status of GSK-3β and the expression of several pro-angiogenic factors, such as human ANG-1, ANG-2, and VEGF to elucidate the relationship between *in vitro* angiogenesis and the endocannabinoid system receptors.

## Materials and Methods

***Cell line and culture conditions***

Human Cerebral Microvascular Endothelial Cells (hBMECs)(CRL-3245™) were acquired from ATCC and were cultured in DMEM/F12 media supplemented with 10% Fetal Bovine Serum (FBS), 1% of antibiotic concentration (penicillin and streptomycin), rhEGF, hydrocortisone, heparin sulfate, bovine brain extract, L-glutamine, and ascorbic acid (PCS-110-040™) (ATCC, Manassas, VA). Incubation and cell growth had taken place under premium conditions at 37 °C in 95% air and 5% CO_2_ incubators. Cell passaging was performed on a ratio of 1:4 (21).

***Drug treatment***

The drug (R)-5-Fluoro-ADB was ordered from Cayman Chemical Company (16603). The stock solution was diluted to prepare five different concentrations (0.0001 μM, 0.001 μM, 0.01 μM, 0.1 μM, and 1 μM) in addition to the control. 

***MTT assay***

The effect of drug treatment on viability was assessed using MTT assay, which relies on the reduction of tetrazolium particles to formazan crystals. The cells were harvested and counted with a total of 5 x 10^3 ^hBMECs per well seeded on a 96-well plate and incubated for 24 hr. Afterward, (R)-5-Fluoro-ADB ranging between 0.0001 μM to 1 μM was added into the cells and incubated for 24 hr. Subsequently, the drug was discarded and replaced with serum-free media containing MTT solution. Each plate was incubated for 4 hr before adding DMSO to each well as a final step, changing the yellow color of the MTT into purple due to the reduction of its particles into formazan. Prior to reading the results, the plates were shaken for 15 min, and the absorbance was then measured using an ELISA reader at 570 nm (21).

***In vitro wound-healing assay***

The endothelial cell migration rate was evaluated by performing an *in vitro* wound-healing assay. The cells were grown in a 12-well plate until they reached approximately 90% to 100% confluency. After incubation, a scratch in the cell monolayer was introduced using a 1000 μl pipette tip, then washed gently using phosphate buffer saline to ensure all detached cells were removed. Afterward, media containing (R)-5-Fluoro-ADB with different concentrations (0.0001 μM, 0.001 μM, 0.01 μM, 0.1 μM, and 1 μM) and control were added to the 12-well plate in triplicate and incubated for 24 hr. Microscopic images were taken at the time the wound was inflicted on the cells monolayer (baseline) and after 24-hour incubation with the drug. All measurements were determined by using the ImageJ software(22).

***In vitro tube formation assay***

The potential to enhance angiogenesis was evaluated through an *in vitro* tube formation assay. Frozen Reduced Growth Factor Basement Membrane Matrix (BMM) (A1413302) was bought from Thermos Fisher Scientific and thawed at 4 °C overnight. 50 ul of BMM were polymerized for 30 min at 37 °C after being added on a 96-well plate. Cell suspension containing 2 x 10^4^ hBMECs in a mixture of growth serum-free media and (R)-5-Fluoro-ADB for three different concentrations (0.0001 μM, 0.01 μM, and 1 μM) was added to the BMM-coated wells and incubated for 24 hr. The results were assessed by capturing microscopic images of the formed tube-like structures created by the cells. Various angiogenic measurements, including the number of tube-like structures, branching points, loop structures, and the total length of each tube, were determined (21). 

***RNA extraction and real-time PCR analysis***

RNA extraction from hBMECs was performed using the total RNA purification kit (PP-210L) (Jena Bioscience, Munich, Germany), following the manufacturer’s recommended protocol. The concentration and purity of the purified RNA samples were measured using a Nano-drop device ND-1000 (Bio Drop, UK). The purified RNA samples were reverse-transcribed into cDNA. The reverse transcription and amplification of the three target genes (VEGF, ANG-1, ANG-2) were performed using the SOLIscript 1-step SolisGreen kit (08-63-00250) The specific reverse transcription and amplification conditions are detailed in Table S1. The primers employed in this study for the proangiogenic genes, along with beta-actin, the reference gene, were chosen based on a previously published article (23). 

***Western blotting***

Western blotting was utilized to assess the protein expression levels in hBMECs. Briefly, the cells were lysed using a mixture of RIPA lysis buffer (ab156034; Abcam) and phosphate-protease inhibitors (A32959; Thermo Fisher Scientific). Then, the protein concentration of each sample was determined using a DC Protein Assay Kit II (#5000112, BioRad, Benicia, CA, US). A total of 20 µg of proteins was loaded onto a sodium dodecyl sulfate-polyacrylamide gel electrophoresis (SDS-PAGE) and transferred to a polyvinylidene fluoride (PVDF) membrane. Each membrane was then blocked using 2% Bovine Serum Albumin (BSA), followed by incubation with the following primary antibodies: anti- anti-β-Actin (4967S; 1:1000; Cell Signaling Technology), anti-ANG-1 (ab94684; 1:500, Abcam), anti-ANG-1 (ab94684; 1:500, Abcam), anti-VEGF-A (ab46154; 1:500, Abcam), anti-Total-GSK-3β (PA5-95845; 1:1000, ThermoFisher), and anti-Ser9-p-GSK-3β (9336S; 1:500, Cell Signaling Technology). The primary antibodies were allowed to incubate with the membranes overnight at a temperature of 4 °C. Finally, HRP-conjugated secondary antibodies were incubated, and the chemiluminescence signals were detected. All images were analyzed using ImageJ Software (24). 

***Enzyme-linked immunosorbent assay (ELISA)***

ELISA was employed to evaluate the quantities of proteins secreted by the cells into the culture media. hBMECs were incubated with (R)-5-Fluoro-ADB for 24 hr before being collected, and then the media was centrifuged for 10 min at 10,000 rpm. Human Angiopoietin 1 ELISA Kit (ab99972), Human Angiopoietin 2 ELISA Kit (ab99971), and Human VEGF ELISA Kit (ab100662). were purchased from Abcam, and all steps were carried out in accordance with the instructions outlined in the kits’ protocols.

***Statistical analysis***

Statistical analysis was performed on the data using one-way ANOVA followed by a Turkey *post-hoc* test using GraphPad Prism (version 9.0.0 GraphPad Software, La Jolla, CA, USA). Statistical significance was attributed to results exhibiting a *P*-value below 0.05.

## Results

***Synthetic cannabinoid (R)-5-fluoro-ADB increases cell viability of hBMECs***

**Figure 2:**
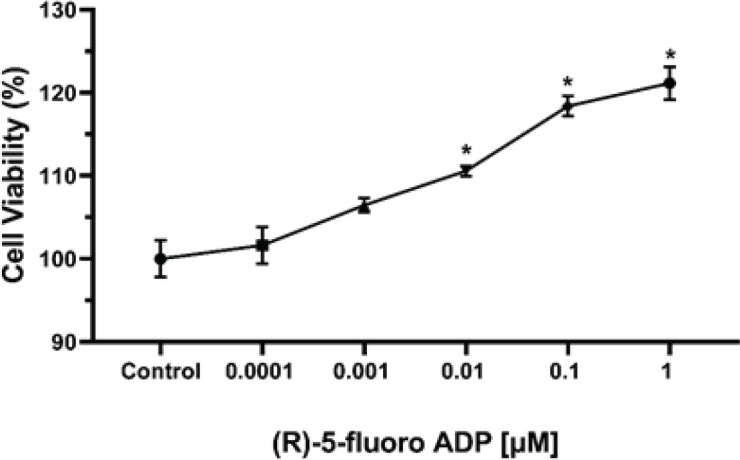
Synthetic cannabinoid (R)-5-Fluoro-ADB has elevated the proliferation of hBMECs, as demonstrated by the MTT viability assay

Cell viability following exposure to the synthetic cannabinoid (R)-5-Fluoro-ADB was assessed using the MTT assay. The results demonstrated a gradual increase in the viability of hBMECs in the treated cells (Figure 2). Significantly higher viability was observed in (R)-5-Fluoro-ADB-treated cells at concentrations ranging between 0.01 μM-1 μM with *P*-values of less than 0.05 (*P-*value = 0.0027 for 0.01 μM, *P-*value*<*0.0001 for both 0.1 μM and 1 μM).

***Synthetic cannabinoid (R)-5-Fluoro-ADB promotes the cell migration rate of hBMECs***

**Figure 3:**
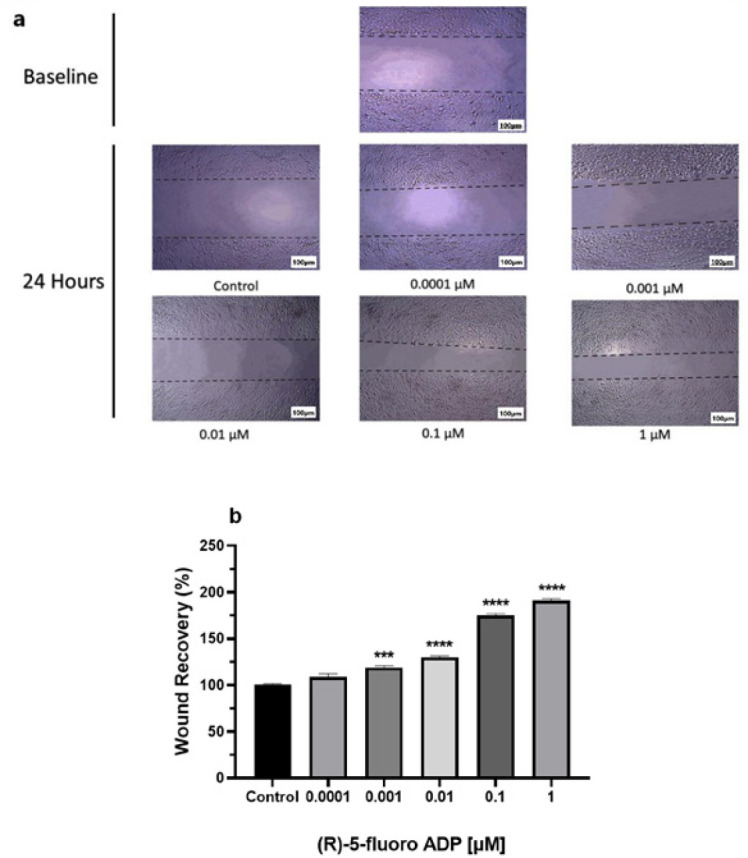
Cell migration was significantly increased through wound-healing assay

 Endothelial cell migration is a crucial and essential step to initiate angiogenesis. The migration rate of hBMECs has elevated at different concentrations of (R)-5-Fluoro-ADB. The microscopic images also indicate a decrease in the size of the wound inflicted on the cell monolayer (Figure 3a). A significant increase has been demonstrated in 0.001 μM (*P-*value* = 0.0343*), 0.01 μM, 0.1 μM, and 1 μM (*P<0.0001*) (R)-5-Fluoro-ADB compared to control (Figure 3b).

***Synthetic cannabinoid (R)-5-fluoro-ADB enhances the tube-like structure formation capacity of hBMECs***

**Figure 4:**
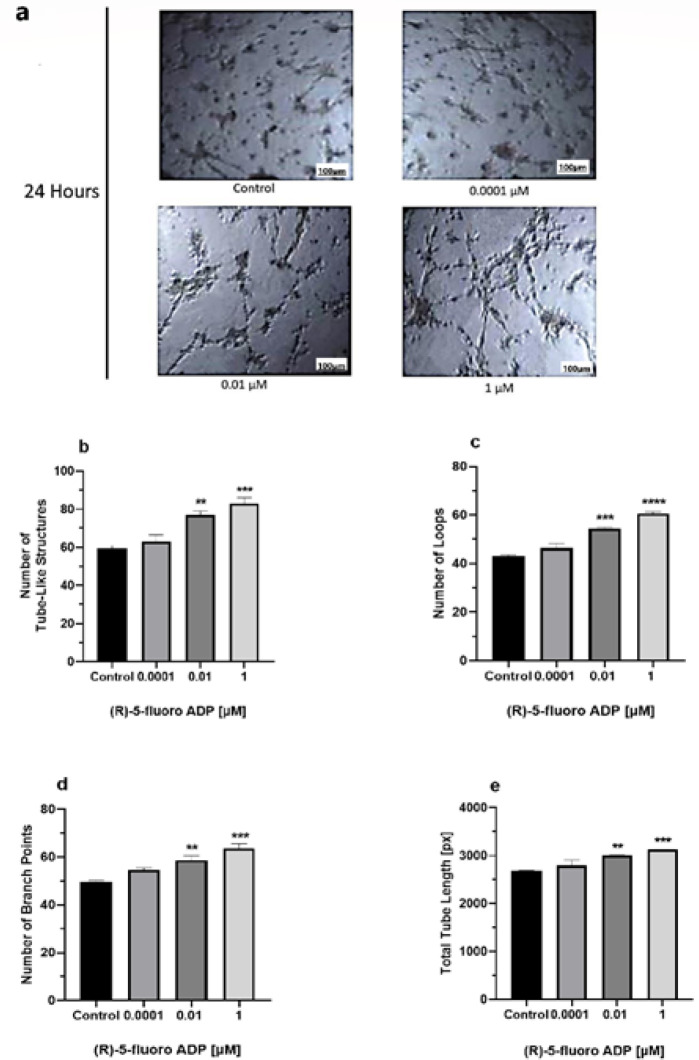
Angiogenic capacity of hBMECs increased after treatment with (R)-5-Fluoro-ADB

The angiogenic potential of hBMECs was assessed through an *in vitro* tube formation assay. The assay was performed using three concentrations (0.0001 μM, 0.01 μM, and 1 μM). Various angiogenic characteristics, encompassing metrics such as the number of tube-like formations, loops, branches, and tube length, were examined to evaluate the capacity for angiogenesis. A significant increase in the angiogenic capacity was observed at 0.01 μM and 1 μM (R)-5-Fluoro-ADB in all angiogenic parameters (Figure 4a-e).

***mRNA expression of ANG-1, ANG-2, and VEGF has increased after treatment with the synthetic cannabinoid (R)-5-Fluoro-ADB***

**Figure 5:**
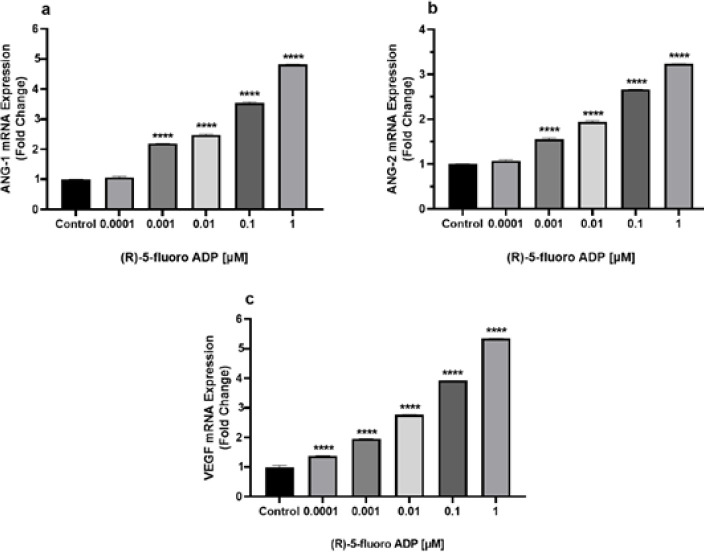
Real-time qPCR displayed a significant increase in mRNA expression of several angiogenesis-related genes

Real-time PCR was performed to evaluate gene expression in hBMECs after (R)-5-Fluoro-ADB treatment. The PCR analysis unveiled alterations in the fold change of the angiogenesis-related mRNA expression. The mRNA levels of ANG-1 showed an approximately 2.2-fold increase at 0.001 μM, 2.5-fold increase at 0.01 μM, 3.5-fold increase at 0.1 μM, and 4.8-fold increase at 1 μM (*P*<0.0001) (Figure 5a). Correspondingly, the levels of ANG-2 mRNA were increased by 1.6-fold at 0.001 μM, 1.9-fold at 0.01 μM, 2.7-fold at 0.1 μM, and 3.2-fold at 1 μM (*P*<0.0001) compared to control (Figure 5b). Furthermore, the mRNA expression of VEGF significantly elevated after treatment with (R)-5-Fluoro-ADB, showing an increase by 1.4-fold at 0.0001 μM, 2-fold at 0.001 μM, 2.8-fold at 0.01 μM, 4-fold at 0.1 μM, and 5.4-fold at 1 μM (*P*<0.0001) compared to control.

***Synthetic cannabinoid (R)-5-fluoro-ADB increases intracellular protein expression of Ser9-p-GSK-3β, ANG1, ANG-2, and VEGF***

**Figure 6:**
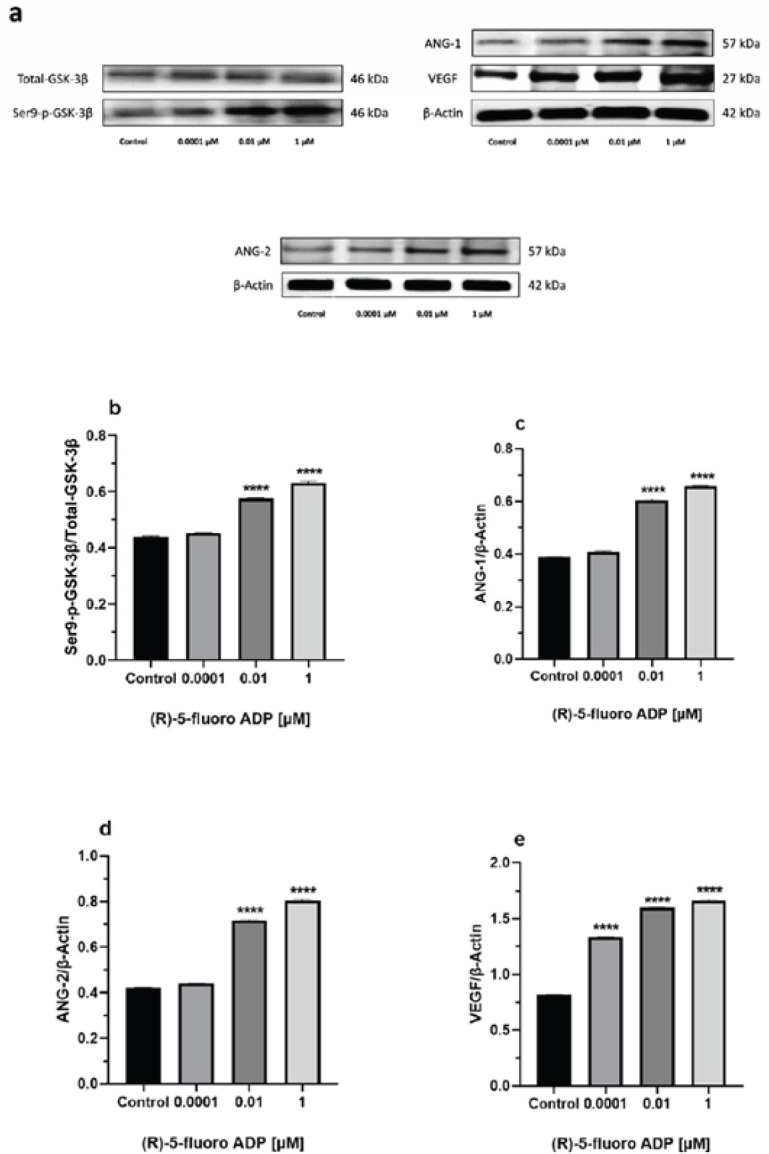
Intracellular protein expression rates of p-Ser-9-GSK-3β/GSK-3β, ANG-1, ANG-2, and VEGF in hBMECs

Western blotting was applied to determine protein concentration and expression in the cells. Four angiogenic-related proteins including Ser9-p-GSK-3β, ANG-1, ANG-2, and VEGF were analyzed under three concentrations of (R)-5-Fluoro-ADB (0.0001 μM, 0.01 μM, and 1 μM). Specific bands of Ser9-p-GSK-3β (~46 kD), ANG-1 (~57 kD), ANG-2 (~57 kD), and VEGF (~27 kD) were detected in (R)-5-Fluoro-ADB-treated hBMECs cells as well as the control (Figure 6a). The findings revealed a significant increase in the expression of these proteins at 0.01 μM and 1 μM (R)-5-Fluoro-ADB, as compared to the control (Figure 6b-e). 

***Concentration of secreted ANG-1, ANG-2, and VEGF is increased by the synthetic cannabinoid (R)-5-fluoro-ADB***

**Figure 7:**
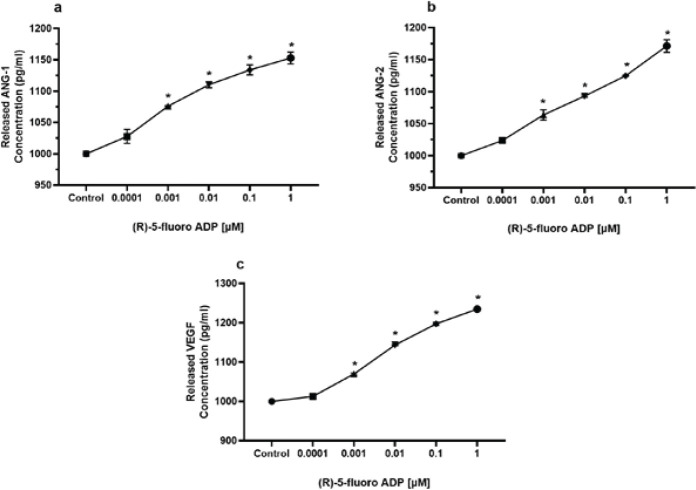
Release of ANG-1, ANG-2, and VEGF has significantly stimulated after incubation with (R)-5-Fluoro-ADB

ELISA was employed to measure the concentrations of ANG-1, ANG-2, and VEGF proteins secreted by cells in the media. Protein secretion was investigated at five different concentrations of (R)-5-Fluoro-ADB ranging between 0.0001 μM and 1 μM. The findings represented a significant increase in protein secretion levels for the four highest concentrations (0.001 μM–1 μM) in all angiogenic-related proteins (Figure 7a-c).

A total of 5 x 103 hBMECs in 100 µl of DMEM/F12 media were cultured in each well of a 96-well plate for 24 hr. Subsequently, the hBMECs were incubated with (R)-5-Fluoro-ADB for 24 hr. The cells were treated with MTT solution for 4 hr, and the absorbance at 570 nm was measured using an ELISA reader for each well. The data were presented as mean ± SEM. (*) indicates *P*<0.05. (n = 3).

(a) Representative microscopic images illustrate the cell monolayer at baseline (zero time) and after 24 hr of incubation with different concentrations of (R)-5-Fluoro-ADB. (b) Quantitative analysis of the migration data showed a significant gradual increase at different concentrations of (R)-5-Fluoro-ADB have been observed. (***) indicates *P*-value<0.001. (****) indicates *P*-value<0.0001. (n = 3).

(a) Representative microscopic images depict the formation of tube-like structures after 24 hr of incubation with (R)-5-Fluoro-ADB. A notable rise was detected at different concentrations of (R)-5-Fluoro-ADB. The investigated angiogenic parameters were as follows: (b) tubular structures, (c) tube length, (d) number of branches(e) loops. (**) indicates *P*-value<0.001. (***) indicates *P*-value<0.001. (****) indicates *P*<0.0001. (n = 3).

Quantitative analysis of the mRNA expression rate of (a) ANG-1, (b) ANG-2, and (c) VEGF in HBMECs after treatment with (R)-5-Fluoro-ADB. (****) indicates *P*-value<0.0001. (n = 3).

Total proteins were isolated from hBMECs after treatment with 0.0001 μM, 0.01 μM, 1 μM (R)-5-Fluoro-ADB, and control. The protein expression was detected by western blot analysis using targeted primary antibodies. (a) The resulting representative blot. Densitometric analysis of (b) Ser9-p-GSK-3β/Total-GSK-3β, (c) ANG-1, (d) ANG-2, and (e) VEGF was performed using ImageJ software. (****) indicated *P*<0.0001. (n = 3).

By using ELISA, the released levels of (a) ANG-1, (b) ANG-2, and (c) VEGF were investigated. (*) indicated *P*<0.0001. (n = 3).

## Discussion

Synthetic cannabinoids (SCs) were initially developed to mimic the impacts and interactions of Phytocannabinoids, aiding in the study of the reactions that occur when these compounds bind to the cannabinoid receptors on cells. Unfortunately, these compounds were subsequently misused as addictive substances, resulting in unforeseen adverse effects and, in certain instances, even fatal overdoses. However, extensive research over the years has shown that SCs have a strong correlation with various cellular and chemical activities in the body including angiogenesis, which is the focus of this investigation. The cannabinoid receptors CB1 and CB2 are recognized as two of the most prevalent receptors in the body and brain cells, influencing intracellular signals within the brain and the human nervous system (25). The synthetic cannabinoid (R)-5-Fluoro-ADB is considered a potent agonist to both CB1 and CB2 (17). This study, has demonstrated significant effects on the angiogenesis of brain cells, as well as influencing various cellular processes such as protein and mRNA expression within the cells.

Numerous studies conducted in recent decades have focused on investigating the effects of SCs on various types of human cells. SCs have the potential to impact intracellular processes, which could have effects that might lead to either stimulation or inhibition of cell viability. An investigation was carried out to assess the influence of JWH-018 on the viability of SH-SY5Y neuronal cells, with the findings revealing no noteworthy effect on these cells (26). However, another study investigating the impact of the synthetic cannabinoid CB83 on human HT-29 colorectal adenocarcinoma cells clearly demonstrated a significant decrease in cell viability (27). In the current study, the viability of hBMECs treated with (R)-5-Fluoro-ADB was assessed using the viability MTT assay, and the results revealed a significant increase in cell viability. These findings are consistent with a previous study that demonstrated enhanced proliferative rates and reduced toxicity effects on hBMECs when exposed to the synthetic cannabinoid XLR-11 (21).

Cell migration is a fundamental characteristic of angiogenesis, cell development, cancer cell metastasis, and inflammation. A living cell is expected to possess the ability to migrate and thrive within tissues and organs, contributing to physiological and cellular processes such as the immune response (28). After exposure to the synthetic cannabinoid XLR-11, there was a significant enhancement in both migration and brain angiogenesis rates (21). Moreover, the knockdown of the CB1 receptors, the main receptors of (R)-5-Fluoro-ADB, has impaired migration and the capacity to form tubular structures (29). On the other hand, anti-angiogenic effects have been observed after administration of different types of SCs. For instance, WIN 55,212-2, a synthetic cannabinoid, has reduced angiogenesis and proliferation through MAPK/Akt-mediated apoptosis signaling in human endometriotic cells (30). Furthermore, antimetastatic and antitumor impacts have been observed in colon cancer and melanoma after exposure to the synthetic cannabinoid URB447 (31). In the current study, the migration rate of HBMECs was evaluated using an *in vitro* wound healing assay after incubation with (R)-5-Fluoro-ADB. The results demonstrated a significant increase in migration rate, with the inflicted wound on the cell monolayer narrowing at a faster rate with increasing drug concentration. Moreover, the angiogenesis capacity induced by (R)-5-Fluoro-ADB was validated using an *in vitro* tube formation assay, revealing a clear increase in all investigated angiogenic parameters. These findings suggest that (R)-5-Fluoro-ADB could be a potent angiogenic drug. Consequently, the increased rates of brain angiogenesis and proliferation directly correlate with the activation of cannabinoid receptors, indicating that (R)-5-Fluoro-ADB holds potential for further research and possible therapeutic applications in angiogenesis-related diseases.

Angiogenesis regulation involves a multitude of signaling pathways that rely on protein-receptor interactions. Among these pathways, the Angiopoietin family consists of ANG-1 and ANG-2, both of which bind to the tyrosine kinase Tie2 receptor (7). Interestingly, these exhibit contradictory roles in regulating angiogenesis, with ANG-1 promoting vascular formation and protection, while ANG-2 counteracts these effects and disrupts angiogenesis (7). ANG-2 triggers a proangiogenic response by promoting endothelial cell migration, proliferation, and the emergence of new branches, particularly in the presence of VEGF (32). Upon treating brain endothelial cells with (R)-5-Fluoro-ADB, we noticed an increase in the expression of both ANG-1 and ANG-2. These findings draw attention to the potential biological function of ANG-1 and ANG-2 in promoting brain angiogenesis. Furthermore, it underscores their relationship with cannabinoid receptors, particularly in the presence of VEGF.

VEGF participates in most angiogenic signaling processes when binding to vascular endothelial growth factor receptors (VEGFRs) (33). VEGF is involved in several vascular functions such as the development of the blood vessel lumen and inducing the proliferation of endothelial cells. VEGF also plays an essential role in endothelial cell migration (6). Moreover, the VEGF protein family was found to regulate embryonic, as well as tumor angiogenesis. Inhibition of VEGFR signaling by anti-VEGF drugs has now become a target for cancer therapy (33). There has been a suggestion that cannabinoid receptor activation significantly reduces the levels of VEGF in various cancer cell lines (34). Numerous studies have established that receptor behavior exhibits variations based on the specific characteristics and types of cells involved. In the present work, we discovered that incubation with (R)-5-Fluoro-ADB substantially improved upon the activation of cannabinoid receptors in the HBMECs. Our results suggest that the proangiogenic factor VEGF may have a crucial role in facilitating the angiogenesis associated with cannabinoid receptor activation in the brain.

GSK-3β is a widely recognized protein involved in various processes throughout the human body, including neurogenesis and neuronal migration. GSK-3β was found to be regulated by transient alterations in calcium levels intracellularly and a calcium-dependent tyrosine kinase (35, 36). Moreover, GSK-3β is highly expressed in the developing brain within neurons, but gradually decreases throughout human life, reaching its lowest concentration in old age (10). GSK-3β becomes activated through autophosphorylation at Tyr216, whereas phosphorylation at Ser9 leads to its inactivation (37). Recent research has indicated that GSK-3 may be a major target for controlling angiogenesis. GSK-3 may be a potential strategy for treating human glioma cells which reveals the critical function of GSK-3 in the regulation of angiogenesis (38). Moreover, angiogenesis is induced by the pathway GSK-3/-catenin by triggering VEGFA in endothelial cells. Inhibiting GSK-3 signaling can potentially enhance angiogenesis by facilitating the release of numerous angiogenic proteins from endothelial cells (39). A study has demonstrated that the synthetic cannabinoid HU-210 enhances the proliferation rate of cerebellar granule cell precursors (GCPs) by activating CB1 receptors. The administration of HU-210 has resulted in augmentation of Ser9-p-GSK-3β phosphorylation, indicating the engagement of the phosphorylated Ser9-p-GSK-3β pathway in the management and modulation of GCP proliferation (40). We discovered that after treatment with (R)-5-Fluoro-ADB, the levels of Ser9-p-GSK-3 phosphorylation, and subsequently GSK-3 inactivation, upon stimulation of cannabinoid receptors, were noticeably enhanced in the hBMECs. This implication suggests that GSK-3β might participate in intracellular pathways subsequent to cannabinoid receptor activation, potentially playing a role in brain angiogenesis.

We have demonstrated the proangiogenic impact of the (R)-5-Fluoro-ADB on brain endothelial cells and examined the implications of these findings for the angiogenesis process. However, it is important to acknowledge certain limitations in this research. Vascular formation is a complex process regulated by numerous proteins that were not investigated in this study, including fibroblast growth factors (FGF), platelet-derived growth factors (PDGFs), and the NOTCH and WNT signaling pathways, among others. It is essential to explore the expression profile of these proteins and their association with the activation of cannabinoid receptors. Additionally, investigating other intracellular proteins, such as β-catenin and Akt, which exhibit correlations with GSK-3β, holds considerable importance.

## Conclusion

Every year, new synthetic cannabinoids (SCs) are discovered in drug abusers, as they are developed to evade regulation and exert more potent effects on users. To the best of our knowledge, this is the first study performed on the consequences of (R)-5-fluoro-ADB on brain angiogenesis *in vitro*. Treatment of hBMECs with the synthetic cannabinoid (R)-5-Fluoro-ADB improves the endothelial cells’ viability and vascular formation capacity at moderate and high concentrations, indicating that (R)-5-Fluoro-ADB stimulates brain angiogenesis. Furthermore, Ser9-p-GSK-3β, ANG-1, ANG-2, and VEGF have mediated the induced-angiogenic signals in the brain endothelial cells. The results reveal additional details about the influence of SCs on brain endothelial cells, which could help with the discovery of novel therapeutics approaches to control and regulate several angiogenesis-related diseases.

## Authors’ Contributions

LN A and SZ A designed the experiments; LN A and SZ A performed experiments and collected data; LN A, SZ A, and IY K discussed the results and strategy; LN A, SZ A, IY K, and MA A supervised, directed, and managed the study; LN A, SZ A, IYK, and MA A approved the final version to be published.

## Conflicts of Interest

The authors declare no conflict of interest, financial or otherwise. 
